# Blood‒Brain Barrier Pathology and CNS Outcomes in *Streptococcus pneumoniae* Meningitis

**DOI:** 10.3390/ijms19113555

**Published:** 2018-11-11

**Authors:** Belinda Yau, Nicholas H. Hunt, Andrew J. Mitchell, Lay Khoon Too

**Affiliations:** 1 Molecular Immunopathology Unit, Bosch Institute and School of Medical Sciences, University of Sydney, Sydney 2006, Australia; nicholas.hunt@sydney.edu.au (N.H.H.); laykhoon.too@sydney.edu.au (L.K.T.); 2 Materials Characterisation and Fabrication Platform, Department of Chemical Engineering, University of Melbourne, Melbourne 3010, Australia; andrew.mitchell1@unimelb.edu.au

**Keywords:** *Streptococcus*, pneumonia, pneumococcal, meningitis, blood‒brain barrier, infection, inflammation

## Abstract

*Streptococcus pneumoniae* is a major meningitis-causing pathogen globally, bringing about significant morbidity and mortality, as well as long-term neurological sequelae in almost half of the survivors. Subsequent to nasopharyngeal colonisation and systemic invasion, translocation across the blood‒brain barrier (BBB) by *S. pneumoniae* is a crucial early step in the pathogenesis of meningitis. The BBB, which normally protects the central nervous system (CNS) from deleterious molecules within the circulation, becomes dysfunctional in *S. pneumoniae* invasion due to the effects of pneumococcal toxins and a heightened host inflammatory environment of cytokines, chemokines and reactive oxygen species intracranially. The bacteria‒host interplay within the CNS likely determines not only the degree of BBB pathological changes, but also host survival and the extent of neurological damage. This review explores the relationship between *S. pneumoniae* bacteria and the host inflammatory response, with an emphasis on the BBB and its roles in CNS protection, as well as both the acute and long-term pathogenesis of meningitis.

## 1. Introduction

Bacterial meningitis is an inflammatory disease of the central nervous system (CNS), diagnosed by the presence of bacteria within the cerebrospinal fluid (CSF). Gram-positive *Streptococcus pneumoniae* is a primary cause of meningitis in the developing world [1], alongside other pathogens such as *Haemophilus influenzae* [2] and *Neisseria meningitides* [1,3]. Pneumococcus-mediated blood‒brain barrier (BBB) breakdown causes acute symptoms that range from fever, headache and neck stiffness, to severe CNS complications including hydrocephalus, brain oedema, intracranial haemorrhage, cerebral venous and arterial complications and seizures that contribute to mortality and long-term disabilities [4]. Mortality rates in human patients are between 20% and 50% [5,6,7], with long-term neurological outcomes such as hearing loss, aphasia, learning impairments and chronic seizures observed in up to 60% of patients after bacterial clearance [8,9,10,11].

Despite continuing advances in vaccines and adjuvant therapies, bacterial meningitis is a persistent health problem because of obstacles that include increasing antibiotic resistance [12,13], serotype replacement [14,15,16] and vaccine failure [17]. Significantly, while research on how *S. pneumoniae* interacts with the brain environment is rapidly evolving, our understanding of the pathogenesis of BBB disruption in pneumococcal meningitis (PM) at the molecular level remains incomplete. This review focuses on *S. pneumoniae*-derived factors that drive CNS invasion via the BBB and addresses the implications of BBB pathology in CNS complications in the acute disease state, as well as neurological sequelae post-recovery.

## 2. The BBB in Acute PM

### 2.1. Structure and Function of the BBB

The BBB is a critical structure protecting against invasion of the CNS by pathogens. It consists of specialist endothelial cells that express highly selective tight junctions, and regulatory and supporting pericytes and astrocytic foot processes along a basal membrane (reviewed in [18]). Additional supporting cells include perivascular macrophages (PVM), resident myeloid cells located within the perivascular space that localise adjacent to cerebral blood vessels and regulate vascular stability [19]. PVM are suggested to be key candidates for communication between the CNS and the periphery [20]. Within the brain, astrocytes support BBB function, mediating endothelial and neuron interactions [21], while microglia can mount an antigen-independent innate immune response by pivoting the balance between anti- and pro-inflammatory macrophages [22]. As a functional barrier of continuous non-fenestrated cells between the circulation and brain interstitial fluid, the BBB serves not only to regulate the passage of ions and molecules, ensuring CNS homeostasis and protection from toxins and pathogenic invaders [23], but also to regulate host immune cell extravasation into the brain parenchyma and thus influence local inflammatory responses [24].

By shielding the CNS from peripheral immune cells and antibodies, the BBB has contributed to what has been classically defined as CNS immune privilege, though our understanding of CNS immune surveillance is still evolving [25,26,27]. Newly discovered lymphatic vessels in the dura mater, the meningeal lymphatic system, have been demonstrated to drain both fluid and immune cells from the subarachnoid space into deep cervical lymph nodes, and may indicate significant roles for these vessels in lymphocyte trafficking and antigen presentation [28]. The presence of CNS lymphatics enables non-pathological movement of leukocytes, such as memory T-lymphocytes, into the CNS, and this is thought to be essential for normal neurological function [29]. T-cell status may also be switched by BBB endothelial cells that act as semi-professional antigen-presenting cells (APC) [30]. As both a physical and immunological barrier, the BBB therefore acts as a key determinant of protective homeostatic surveillance during brain infections [31].

### 2.2. Pneumococcal Transmigration across the BBB into the CNS

**Figure 1:**
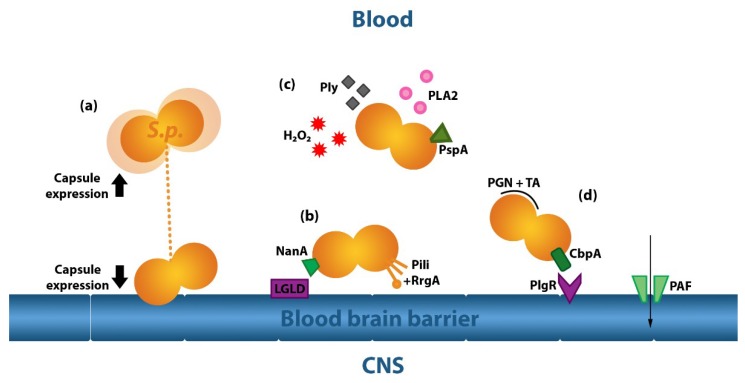
*S. pneumoniae*-mediated CNS invasion and BBB transmigration in PM. (**a**) *S. pneumoniae* regulates high capsule expression, which promotes immune cell evasion, and low capsule expression, which promotes endothelial cell adhesion. Dark arrows represent direction of capsule expression; (**b**) NanA can bind endothelial LGLD, while pili and adhesin RgrA further increase bacterial attachment to endothelial cells, facilitating their BBB translocation; (**c**) secreted proteins include PLA2, which upregulates endothelial adhesins, and hydrogen peroxide and ply, which regulate the overall BBB environment by activating pro-inflammatory host processes leading to cytokine induction and RONS production, as well as directly damaging host endothelial cells; (**d**) PspA interferes with host killing and opsonophagocytosis of *S. pneumoniae* by immune cells, while bacterial cell wall components PGN and TA activate host inflammatory responses through TLR activation. CbpA also inactivates complement pathways, binds human immunoglobin receptor PlgR, and facilitates *S. pneumoniae* translocation across the BBB through the endothelial PAF receptors. Light arrow represents route of *S. pneumoniae* transport. BBB—blood‒brain barrier, CbpA—choline binding protein A, CNS—central nervous system, H_2_O_2_—hydrogen peroxide, LGLD—laminin G-like lectin domain, NanA—neuraminidase A, PAF—platelet-activating factor, PGN—peptidoglycan, PLA2—pneumococcal phospholipase A2, PlgR—poly immunoglobin receptor, ply—pneumolysin, PspA—pneumococcal surface protein A, RONS—reactive oxygen and nitrogen species, RgrA—pilus-related adhesin, TA—teichoic acid.

The key bacterial factors affecting *S. pneumoniae* bloodstream-to-CNS invasion across the BBB are summarised in Table 1 and illustrated in Figure 1.

Colonisation of the intranasal cavity by *S. pneumoniae* is the first step to PM pathogenesis. The bacterium is inhaled through airborne droplets and colonises the mucosal surfaces of the nasopharynx. Asymptomatic nasal carriage of *S. pneumoniae* occurs in almost 30% of all individuals [32,33,34], with higher rates observed in children and neonates [35]. Transmission between humans in close contact means that communities often share *S. pneumoniae* serotype profiles, and this may account for variations in population susceptibility to invasive disease [35,36]. From the nasopharynx, *S. pneumoniae* can progress to the inner ear cavities, the lungs or invade the intravascular space within tissue to access the bloodstream—causing otitis media, pneumonia, or sepsis, respectively [37]. Once *S. pneumoniae* becomes blood-borne, meningitis is preceded by invasion of the CNS through the BBB or blood‒CSF barrier [38], though olfactory neuron invasion also has been observed [39].

We now will discuss key virulence factors that lend advantages to *S. pneumoniae* in blood-to-brain parenchyma invasion, some of which are common to other meningitis-causing pathogens [40]: the pneumococcal capsule, bacterial surface proteins, and secreted proteins such as pneumolysin.

#### 2.2.1. The Pneumococcal Capsule

The pneumococcal capsule, a 200–400 nm thick polysaccharide wall that encompasses the exterior cell wall of *S. pneumoniae*, is a vital regulator of the bacterium’s invasive capacity. Clinical isolates of *S. pneumoniae* are almost always encapsulated [41], with evidence that systemic dissemination in particular is dependent on maximum capsule expression [42]. High capsule expression enhances immune evasion; encapsulated *S. pneumoniae* display reduced neutrophil extracellular trap adhesion [43], and are more resistant to phagocytosis [44], capable of reducing complement deposition on their surface [45]. However, encapsulation is detrimental to successful colonisation [46], inhibiting binding sites of pneumococcal surface proteins (Psp) adhesion molecules on the *S. pneumoniae* cell wall that are required for epithelial cell binding and transcytosis. Unsurprisingly, successful *S. pneumoniae* variants are most capable of altering capsule expression through quorum sensing and phase regulation, transitioning the capsule from thick to transparent variations [47,48] by modulating biosynthesis of oligosaccharide repeats on the cytoplasmic membrane, encoded at the capsular polysaccharide biosynthesis locus [42]. Evidence from serotype studies suggests that mechanisms of immune evasion (such as phagocytosis resistance) that are mediated through capsule regulation vary across serotypes.

#### 2.2.2. Pneumococcal Proteins

Psp drive successful translocation at both nasopharyngeal/bloodstream and bloodstream/brain boundaries. *S. pneumoniae* pili enable bacterial attachment to endothelial cells [49] through pneumococcal pilus-1 [50]. Pilus-related adhesin (RrgA) binds both host Poly Immunoglobin Receptor (plgR) and platelet endothelial cell adhesion molecule (PECAM-1) to facilitate *S. pneumoniae* translocation across the BBB [51]. Surface neuraminidase A (NanA) can facilitate endothelial binding through the endothelial laminin G-like lectin domain [52]. There is evidence that initial translocation of *S. pneumoniae* at the BBB occurs with adhesion at the vascular endothelium of the subarachnoid vessels, before progression to endothelial cells of the cortex and choroid plexus [38].

Psp are important for bacterial entry into the CNS. Types of Psp known as choline-binding proteins (Cbp) attach to the cell surface of *S. pneumoniae* via phosphorylcholine and teichoic components of the pneumococcal cell wall [36]. Cbp include pneumococcal surface protein A (PspA) and choline-binding protein A (CbpA), which disrupt complement pathways to inhibit phagocytosis by immune cells [53,54,55]. Additionally, PspA increases *S. pneumoniae* resistance to killing by human apo-lactoferrin [56], which works in concert with lysozyme to induce pneumococcal lysis [57]. CbpA also binds to human immunoglobin receptors [58,59], including PlgR [60], as well as platelet-activating factor (PAF) receptors on endothelial cells [38]. As such, CbpA can mediate mucosal invasion, as well as *S. pneumoniae* transport across the BBB [60] through the pneumococcal-PAF complex [38,61]. Downregulated CbpA expression is associated with impaired *S. pneumoniae* colonisation [62]. Furthermore, pneumococcal phospholipase A2 (PLA2), which is a secreted bacterial enzyme that also modulates inflammation [63], is a clinical predictor for PM [64]. PLA2 production is associated with upregulation of adhesion molecules in host vascular endothelial cells [65].

Cell wall components and *S. pneumoniae*-derived enzymes also contribute to virulence. Peptidoglycan and teichoic acid have long been known to activate toll-like receptor (TLR)-mediated inflammation [66], while NanA can alter the viscosity of the mucous environment, cleaving N-acetylneuraminic acid from mucin, glycoproteins, glycolipids and oligosaccharides [67], and exposing host epithelial cells to *S. pneumoniae* contact. Pneumococcal IgA1 protease cleaves protective host secretory IgA [68], hyaluronidase degrades connective tissue extracellular matrix component hyaluronan [69], contributing to increased virulence [70], and hydrogen peroxide production mediated by the pyruvate oxidase (SpxB) gene offers competitive advantage in microbial competition [71].

#### 2.2.3. Pneumolysin

The 53-kDa pore-forming toxin pneumolysin (ply) is a major virulence factor produced by *S. pneumoniae*. Present within the bacterial cytoplasm, it is overrepresented in clinically isolated strains [72] and may either be released during autolysis or actively exported from the cell wall [73]. As its name suggests, pneumolysin is cytolytic. It binds host cell membranes and triggers formation of a pre-pore, puncturing the cell membrane and initiating conformational changes within the host cell to create a mature ply pore [74]. The resulting presence of the mature ply pore in host cells drives protein influx and imbalances in signal transduction [75]. Pneumolysin is also a stimulator of classical complement pathways [75], and of both TLR and the nucleotide-binding oligomerisation domain (Nod)-like receptor (NLR)-activated inflammasome pathways [76,77]. It also activates NADPH oxidase and induces reactive oxygen species production in neutrophils in a manner dependent on pneumococcal autolysin LytA [78].

Ply is also likely to play critical roles not only in the processes of bacterial translocation across the BBB, but also in neuropathology. Ply interferes with brain ependymal cilia [79,80], has direct cytotoxic effects on both epithelial and endothelial cells [81], and triggers microglial and neuronal cell death [82,83]. Ply-induced pore formation also affects glial cells, altering astrocytic cell structure and increasing overall BBB permeability [84]. Clinically, extended ply presence in the CSF correlates with mortality in PM [85]. In experimental PM, mice infected with ply-deficient serotype 2 bacteria were protected from invasive disease [86]. However, we have found that infection with serotype 3 and 4 strains deficient in ply leads to reduced TLR-mediated inflammation at the expense of increased bacterial load [87].

### 2.3. Role of the Host Inflammatory Response in Determining Outcome in PM

**Figure 2:**
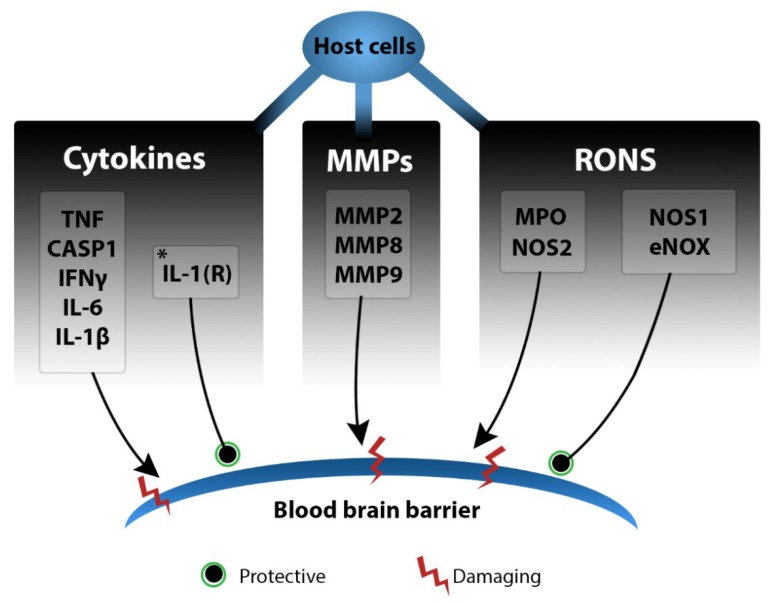
Host-derived components involved in BBB dysfunction. Resident immune cells and infiltrating leukocytes contribute to the cytokine storm, producing proinflammatory mediators TNF, CASP1, IFNγ, IL-1β and IL-6 associated with increase BBB breakdown. However, some aspects of IL-1 signalling through IL-1R may be partially BBB protective. MMP2, 8 and 9 contribute to BBB dysfunction, alongside neutrophil-derived MPO and monocyte-derived NOS2. Endothelial-derived NOS1 is protective of BBB integrity, as is endothelial NADPH oxidase. Arrows to red lines indicate damage, arrows to green circles indicate protection. CASP1—caspase-1, eNOX—endothelial NADPH (nicotinamide adenine dinucleotide phosphate) oxidase, IFNγ—interferon-gamma, IL-1β—interleukin-1-beta, IL-1(R—interleukin-1(receptor), IL-6—interleukin-6, MMP—matrix metalloproteinase, MPO—myeloperoxidase, NOS—nitric oxide synthase, RONS—reactive oxygen and nitrogen species, TNF—tumour necrosis factor.

The immune mediators in PM involved in BBB dysfunction that are discussed in this section are summarised in Table 2, and illustrated in Figure 2.

#### 2.3.1. Microglia and Immune Activation

As the resident macrophages of the brain, microglia are early defence immune system regulators [88,89]. They phagocytose live *S. pneumoniae* [90,91] and are capable of sensing pathogen-associated molecular patterns (PAMPs) through pattern recognition receptors (PRRs) such as TLR and NLR. In response to bacterial invasion, microglia release cytokines and chemokines to instigate a leukocyte infiltration response to bacterial invasion, present antigen to T-cells [92], and may have direct cytotoxic effects on *S. pneumoniae* through antimicrobial peptides [93].

#### 2.3.2. Pattern Recognition Receptors

A range of PRR pathways are triggered within the CNS during pneumococcal meningitis and these influence outcome not only through the host anti-bacterial response but also the associated disruption of CNS function. TLRs are present on glial cells (reviewed in [94]) and have selective capacities to sense a diverse range of PAMPs and danger-associated molecular patterns (DAMPs) of bacterial origin. In PM, a number of virulence factors or pneumococcal proteins can trigger these receptors [95]. For instance, surface-bound TLR2 on glial cells is activated upon recognition of peptidoglycan and lipoteichoic acid in the *S. pneumoniae* cell wall [96,97,98], while ply stimulates TLR4 [76,99]. Endosomal TLR9 responds to *S. pneumoniae* CpG motifs in genomic DNA [100], but requires prior surface recognition and uptake of *S. pneumoniae* into endolysosomes or phagolysosomes [101]. In addition to TLRs, NLRs localise within the cytosol, alongside pyrin and hematopoietic interferon-inducible nuclear antigens with a 200-amino-acid repeat (HIN) domain-containing proteins (PYHIN), to sense intracellular PAMPs and DAMPs (reviewed in [102]). A subfamily of NLRs containing an N-terminal pyrin domain can form multi-protein structures termed inflammasomes, a number of which are sensors of *S. pneumoniae* PAMPs [103]. The inflammasomes consist of the PRR, an adapter protein (typically), and the enzyme caspase-1 (CASP1). In PM, the NLR family pyrin domain containing 3 (NLRP3) inflammasome is activated by ply [104] through extracellular ATP [105]-induced lysosomal disruption and Cathepsin B release [106]. Similarly, the PYHIN protein Absent in Melanoma 2 (AIM2) inflammasome complex responds to cytosolic pneumococcal DNA release from phagolysosomes, which may in turn be dependent upon ply-induced lysis [103,107]. Inflammasome activation ultimately results in CASP1 cleavage of pro-forms of interleukin-1-beta (IL-1β) and interleukin-18 (IL-18) into active releasable forms [108]. Release of active IL-1β contributes to increased inflammation [109], while IL-18 release modulates interferon-gamma (IFNγ)-dependent pathogenesis in PM [110].

#### 2.3.3. Leukocyte Infiltration and the Cytokine Storm

Following PRR-mediated microglial activation, glia-initiated leukocyte infiltration drives the proinflammatory response associated with PM. Local production of interleukin-6 (IL-6), tumour necrosis factor (TNF) and IL-1β from endothelial cells, microglia and astrocytes occurs prior to leukocyte infiltration, with heightened levels of these cytokines characteristic of both clinical [111,112,113,114] and experimental [110,115,116,117,118] PM. It is likely that PVM also have supportive roles in leukocyte transmigration, with PVM depletion being associated with reduced leukocytosis into the subarachnoid space in PM [119].

Upregulation of chemokines in the CNS is characteristic of PM, and these are involved in both leukocyte recruitment and migration. Produced by resident immune cells including microglia, the chemokines chemokine (C-C motif) ligand (CCL)2, CCL3, chemokine (C-X-C motif) ligand(CXCL)8 and CXCL1 regulate neutrophil, monocyte and T-cell chemotaxis, while CXCL1 and CXCL3 are associated with Natural Killer cell recruitment [120]. At the BBB, integrin activation by chemokines such as CXCL12, CCL11 and CCL21 induces leukocyte adhesion [121], with CXCL12 demonstrated to induce both arrest and crawling of T cells, as well as mediate adhesion of monocytes on human vascular endothelial cells in vitro [122]. Mechanisms behind leukocyte diapedesis in the CNS are not well defined, though transmigration of leukocytes occurs either through the paracellular route between endothelial cells or the transcellular route through BBB cells [123], with granulocyte transmigration showing preference for transcellular routes [124].

Neutrophils likely have multifaceted roles in controlling *S. pneumoniae* in the brain. In PM, neutrophils comprise 90% of infiltrating leukocytes [118], and though high white blood cell counts are associated with improved clinical outcomes [125], experimental leukocyte depletion reduces CNS injury and increases survival rates [126]. In contrast, specific neutrophil depletion leads to increased bacterial numbers in the brain and worsened survival in mice [127], and prolonged neutrophil presence in the CNS increases haemorrhage and oedema [128]. In the long term, after PM has been cured by antibiotic treatment, neutrophil-depleted mice display improved behavioural and learning outcomes compared to their non-depleted counterparts [129]. Elucidating the dual protective and harmful roles of leukocytes in CNS infection is crucial to understanding pathogenesis and developing therapies for PM [130].

The presence of leukocytes within the CNS further contributes to the cytokine environment established by resident CNS cells, creating a “cytokine storm”. CSF levels of the archetypal inflammatory cytokines TNF, IL-1β, IFNγ and IL-6 are consistently measurable in clinical PM [116,131,132] and correlate with meningitis mortality [133]. In experimental PM, high intrathecal levels of TNF correspond with increased neutrophil infiltration and BBB breakdown [134], though complete TNF deficiency results in increased mortality [135]. IL-6 gene knockout mice similarly display increased mortality [136], though in this case BBB permeability and brain oedema are diminished [137]. Reduced levels of IL-1β in CASP1 gene knockout mice are associated with improved BBB integrity [138]; however, IL-1 receptor knockout mice were found to have greater BBB invasion, with increased numbers of pneumococci in the CNS [139], indicating that aspects of IL-1 signalling are involved in host protection in PM. IFNγ levels, in particular, correlate with PM in bacterial meningitis caused by other agents [114,135], with increased CSF levels reported in both human patients [114,140] and experimental models [110,118,141,142]. IFNγ activates macrophages and antigen-presenting cells and, along with IL-1β, regulates production of other cytokines [143], making it a critical regulator of the cytokine storm. It is produced by resident CNS cells, infiltrating Natural Killer cells and activated T cells [144] and in PM its production is induced via a pathway involving an inflammasome, IL-12 and IL-18 [110,144]. IFNγ gene knockout mice are protected from mortality in experimental PM and display improved bacterial clearance in the CSF and reduced BBB permeability [110]. Together, these studies highlight the seeming inconsistency and complexity of the cytokine environment in the regulation of BBB integrity and PM pathogenesis.

#### 2.3.4. Reactive Oxygen and Nitrogen Species

Reactive oxygen and nitrogen species (RONS) are released by resident CNS cells, such as microglia and endothelial cells, as well as infiltrating leukocytes during phagocytosis [145], and their levels are elevated in patient CSF and both the CSF and brains of experimental animals with PM [146]. Endothelial NADPH oxidase is protective against BBB disruption in PM [147]. However, RONS also drive multiple aspects of host CNS damage, including BBB breakdown [148]. Upon entry into the CNS, *S. pneumoniae* continues to multiply or undergo autolysis, with either process capable of inducing hydrogen peroxide production, causing cytotoxicity to nearby host cells [149]. Hydrogen peroxide also reacts with host-derived nitric oxide to form peroxynitrite, which in turn is capable of host cell membrane disruption through lipid peroxidation [145], protein carbonyl formation and activation of matrix metalloproteinases [150]. Additionally, hydrogen peroxide conversion to hypochlorous acid by neutrophil-derived myeloperoxidase activates matrix metalloproteinase (MMP)-9, driving BBB breakdown [151]. In PM, treatment with peroxynitrite scavengers alongside antibiotic therapy leads to decreased local IL-1β levels and reduced leukocyte infiltration into the CSF [145]. Similarly, treatment with the hydrogen peroxide scavenger catalase, and superoxide dismutase, reduces brain oedema in PM [152,153].

Nitrite/nitrate and nitric oxide metabolites are observed in the brains of meningitis patients and experimental animals [154], while nitric oxide synthases (NOS) such as NOS2 are specifically linked to BBB breakdown and augmented proinflammatory cytokine profiles in experimental PM [132,155], as well as regulating caspase-3-driven neuronal apoptosis in the hippocampus [156]. Interestingly, endothelial NOS (NOS1) appears to have a protective role, with NOS1 deficiencies associated with increased BBB breakdown, leukocyte infiltration [133] and mortality [157]. In contrast, inducible NOS (NOS2) is produced by infiltrating monocytes and regulated in part by IFNγ in experimental PM [132]. Increased NOS2 expression correlates with increased serum nitrite levels, BBB permeability and protein influx into the brain, with NOS2 deficiency associated with complete BBB protection, alongside reduced oedema, lower concentrations of proinflammatory cytokines in the brain, and lessened mortality [132]. Correspondingly, free radical scavenger treatment that reduced NOS2 levels in the PM brain also correlated with decreased leukocyte infiltration and improved mortality [158].

Overall, RONS play both protective and deleterious roles, and the sites of production and action of these molecules likely determine their impact in PM.

#### 2.3.5. Matrix Metalloproteinases

Matrix metalloproteinases (MMPs), which are zinc-dependent endopeptidases, are secreted by activated leukocytes [159] and are implicated specifically in BBB damage in PM. MMPs degrade the extracellular matrix [160] and MMP8 and MMP9 are measurably increased in the CSF of patients with bacterial meningitis [161], with MMP9 associated with BBB dysfunction and neuronal apoptosis [133,162]. MMP inhibition in conjunction with antibiotic treatment protects from experimental hippocampal injury in PM [162,163] and improves survival [164], with MMP2 and MMP9 single and dual-inhibition reducing BBB breakdown in the hippocampus and/or the cortex [165]. Correspondingly, the metalloproteinase tumour necrosis factor alpha converting enzyme (TACE) is implicated in augmenting MMP release [166], with TACE inhibition being protective against CNS damage, neurological symptoms and mortality in experimental PM [163].

## 3. BBB Disruption and Long-Term Neurological Sequelae in PM

As reviewed above, evasion of host physical and immune barriers allows pneumococci to enter the CNS, which triggers a cascade of inflammatory responses and the recruitment of immune cells to the site. This process leads to a permeable BBB that allows both *S. pneumoniae* and infiltrating leukocytes to further augment the host immune response via multiple positive feedback loops. A well-balanced host immune reaction facilitates complete recovery from PM. However, dysregulated immune responses might occur in many PM cases, which contributes to wide-ranging neurological complications that result in life-long disabilities, including behavioural disorders, cognitive impairments and hearing deficits [167].

In general, dysregulated host inflammatory responses result in two primary catastrophic events—oxidative stress and cytokine storm. These two events are linked to cellular injury and damage, including disrupting the BBB to further trigger long-lasting brain damage. Treatment with antioxidants has beneficial effects against long-term neurological deficiencies in experimental PM. Peroxynitrite scavengers reduce hearing loss [168], while adjuvant treatment with N-acetylcysteine reduces both memory loss and hearing loss [168,169]. In a similar vein, adjuvant administration of matrix metalloproteinase inhibitors in experimental PM reduces damage to BBB and cortex and restores cognitive impairment [164,165], while neuronal damage in the hippocampus has been found to be correlated positively with learning disabilities and cognitive deficits in both human and animal meningitis survivors [170].

The cytokine storm, and its clinical implications for the CNS, have been reviewed recently [171]. Notable pro- and anti-inflammatory mediators involved in driving the pathogenesis of PM, such as IL-6, IL-1β, IFN-γ, IL-10 and transforming growth factor-beta (TGF-β), have been shown to modulate neural progenitor cells’ survival, proliferation and differentiation [172]. Excessive expression of IL-6, TNF and IL-1β—the major cytokines contributing to sickness behaviours during acute PM—may lead to long-lasting sensitisation of neural or endocrine circuits, such as the hypothalamus-pituitary-adrenal (HPA), that modulate emotion, behaviour and cognition [173,174,175]. In experimental PM, acute IL-1β levels correlate with the incidence of neurological sequelae [176], and inversely associate with BBB integrity [138].

Exposure to pathological levels of inflammatory cytokines may also lead to irreversible cellular genetic changes via epigenetic mechanisms, thereby contributing to altered neuro-behavioural functions [177]. In our study [141], we found reduced BBB permeability and cytokine production in mice deficient in IFN-γ compared to their WT counterparts. In the long term, IFNγ gene knockout mice with suppressed immune reactions were shown to survive PM with decreased hippocampal and cortical brain damage, which was linked to improved behavioural disorders and cognitive flexibility. Unlike other gene knockout mouse strains (TLR2/4, IFNγ and NOS2) observed in our study, about 60% of Myeloid differentiation primary response 88 (MyD88) gene knockout mice, which have a substantially attenuated inflammatory response, including reduced leukocytosis and pro-inflammatory cytokine and chemokine production during acute PM, retained their hearing ability as measured by Preyer’s reflex [178].

Altogether, these findings implicate oxidative factors and several cytokines in causing the long-term neurological impacts of PM in survivors of acute disease.

## 4. Concluding Remarks

BBB repair as therapy is currently underutilised. Glucocorticosteroid treatment in multiple sclerosis has been shown to improve BBB integrity and downregulate BBB-compromising effectors such as VEGF [179]. In patients, adjuvant corticosteroid treatment reduced mortality alongside hearing loss and neurological sequelae in adults with PM [180], and dexamethasone used as adjunctive therapy alongside antibiotics reduces CSF levels of MMP9—a previously implicated regulator of BBB damage—as well as overall CNS inflammation and long-term deficits [165,181].

**Figure 3:**
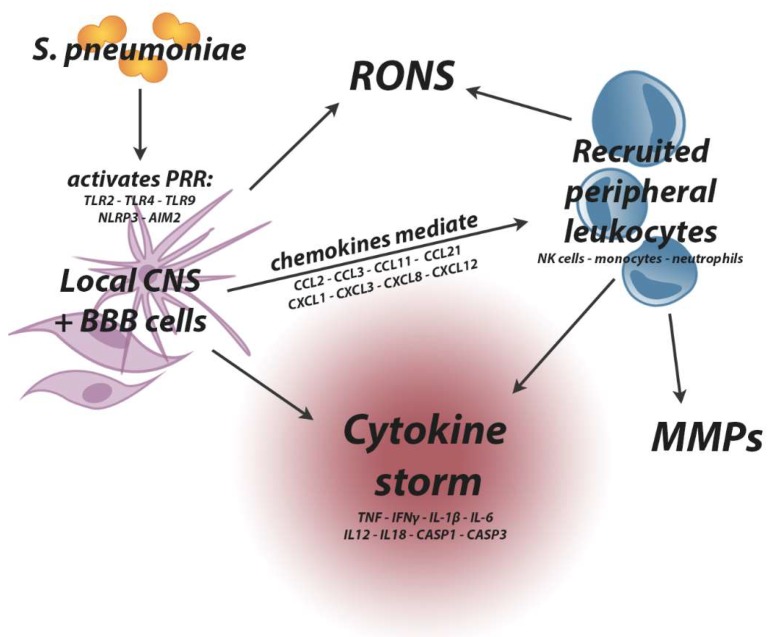
Interactions between *S. pneumoniae,* the brain and the peripheral immune system drives pathogenesis in PM. *S. pneumoniae* activates resident CNS and BBB cells through PRR to initiate the inflammatory response. Chemokines released in the brain mediate recruitment and infiltration of peripheral leukocytes, including neutrophils, monocytes, macrophages and Natural Killer cells, into the brain. Local glial and endothelial cells and recruited immune cells produce RONS and cytokines, while leukocytes also produce MMPs. Black arrows indicate direction of interaction. BBB—blood‒brain barrier, CNS—central nervous system, MMPs—matrix metalloproteinases, PRR—pattern recognition receptors, RONS—reactive oxygen and nitrogen species.

Preserving BBB integrity is key to neurological protection in infectious brain diseases such as bacterial meningitis, as well as non-infectious neurological diseases, including Alzheimer’s disease, epilepsy, ischemic stroke and multiple sclerosis. It is well recognised that the induction of cytokines, oxidative stress, as well as the production of bacterial toxins, compromise BBB integrity in PM, and this is subsequently associated with causing both acute intracranial complications and lasting neurological dysfunction. Figure 3 provides an overview of the known players that drive BBB damage. Our current review of the implications of BBB pathology in PM pathogenesis identifies a shortfall in the field. The measurement of BBB disruption is uncommon in meningitis studies, and CNS leukocytosis and/or heightened pro-inflammatory cytokines and chemokines are generally an accepted proxy for BBB breakdown. The findings reviewed herein hopefully provide insight into BBB maintenance as a potential therapeutic target and the importance of addressing the BBB in the understanding of PM pathogenesis.

## Figures and Tables

**Table 1:** *S. pneumoniae*-derived virulence factors and their modes of action in contributing to CNS invasion, and BBB transmigration and dysfunction in PM.

	Virulence Factor	Mode(s) of Action	Reference(s)
Capsule	Thick polysaccharide capsule	Reduce neutrophil extracellular trapping	[43]
		Reduce phagocytosis	[44]
		Reduce complement deposition	[45]
	Thin polysaccharide capsule	Expose pneumococcal surface protein binding sites	[43,44,45]
Pili	Pneumococcal pilus-1	Increase attachment to endothelial cells	[51]
	Pilus adhesin RrgA	Facilitates BBB translocation through pIgR and PECAM-1 binding	[51]
Choline binding proteins	Pneumococcal surface protein A (PspA)	Interferes with complement factor B	[53]
		Inhibits human apo-lactoferrin activity	[56]
	Choline-binding protein A (CbpA)	Inactivates C3b through complementary factor H binding	[54,55]
		Facilitates BBB translocation through plgR and PAF receptors on endothelial cells	[38,51,60]
Cell wall	Peptidoglycan	Activates host TLR, increasing inflammation	[66]
	Teichoic Acid	Binds choline-binding proteins to pneumococcal cell wall	[36]
		Activates host TLR, increasing inflammation	[66]
Pneumococcal surface proteins	Neuraminidase A	Cleaves N-acetylneuraminic acid	[67]
		Facilitates endothelial binding through LGLD	[52]
Secreted	Pneumococcal IgA protease	Cleaves host secretory IgA	[68]
	Hyaluronidase	Degrades hyaluronan	[69]
	Pneumococcal phospholipase A2 (PLA2)	Increases inflammation	[63]
		Associated with upregulation of endothelial cell adhesins	[65]
	Hydrogen peroxide	Kills competing microbes	[71]
	Pneumolysin	Cytolytic through ply pore formation to epithelial, endothelial and glial cells	[74,81,82,83,84]
		Stimulates complement pathways	[75]
		Activates TLR and NLR inflammasome pathways	[76,77]
		Activates NADPH oxidase	[78]
		Activates ROS production in neutrophils	[78]
		Disrupts ependymal cilia	[79,80]

BBB—blood‒brain barrier, IgA—immunoglobin A, NADPH—nicotinamide adenine dinucleotide phosphate, NLR—NOD-like receptor, PAF—platelet-activating factor, PECAM-1—platelet endothelial cell adhesion molecule-1, plgR—poly immunoglobin receptor, ply—pneumolysin, Psp—pneumococcal surface protein, RgrA—pilus-related adhesin, ROS—reactive oxygen species, TLR—toll-like receptor.

**Table 2:** Host-derived mediators associated with immune modulation and BBB permeability.

	Mediator	Associated Immune Consequence in PM	Reference(s)
**Chemokines**	CCL2	Monocyte, neutrophil and T-cell recruitment	[120]
	CCL3		
	CXCL8		
	CXCL1	Monocyte, neutrophil and T-cell recruitmentNatural Killer cell recruitment	
	CXCL3	Natural Killer cell recruitment	
	CXCL12	Activates endothelial cell integrins to induce leukocyte adhesion	[121,122]
	CCL11		
	CCL21		
**Cytokines**	TNF	Induces neutrophil infiltrationIncreased BBB breakdown	[134]
	IL-1β	Regulates inflammatory cytokines	[143]
	IL-6	Increased BBB permeability	[137]
	IFN-γ	Activates macrophages and T-cellsRegulates inflammatory cytokinesIncreases BBB permeability	[110,143,144]
	CASP1	Regulates IL-1β	[138]
	CASP3	Increases hippocampal apoptosis	[156]
**RONS**	H_2_O_2_	Increases neuronal damage	[149]
		Increases lipid peroxidation	[145]
		Increases and activates MMPs	[150]
		Increases brain oedema	[152,153]
	eNOX	Protects against BBB damage	[147]
	NOS1	Reduces leukocyte infiltration into the CNSProtects against BBB damage	[133]
	NOS2	Increases serum nitriteIncreases leukocyte infiltration into the CNSIncreases BBB permeability	[132,158]
**MMPs**	MMP2	Increases BBB permeability	[167]
	MMP9	Increases BBB permeabilityIncreases neuronal apoptosis	[133,162,165]

BBB—blood‒brain barrier, CCL—C-C motif chemokine, CXCL—C-X-C motif chemokine, CASP1—caspase 1, CASP3—caspase 3, CNS—central nervous system, eNOX endothelial NADH oxidase, ICAM-1—intercellular adhesion molecule 1, IFNγ—interferon-gamma, IL-1β—interleukin-1-beta, IL-6—interleukin-6, MMP—matrix metalloproteinase, NOS—nitric oxide synthase, PM—pneumococcal meningitis, RONS—reactive oxygen and nitrogen species, TNF—tumour necrosis factor, VCAM-1—vascular cell adhesion molecule 1.

## Abbreviations

APC	Antigen-presenting cells
ASC	apoptosis-associated speck-like protein containing a CARD domain
BBB	blood‒brain barrier
CASP1	caspase-1
Cbp	choline-binding proteins
CNS	central nervous system
CSF	cerebrospinal fluid
DAMP	danger-associated molecular patterns
HPA	hypothalamus‒pituitary‒adrenal
IFNγ	interferon-gamma
IL-1β	interleukin-1-beta
IL-6	interleukin-6
IL-12	interleukin-12
IL-18	interleukin-18
LytA	pneumococcal autolysin
MMP	matrix metalloproteinase
MyD88	myeloid differentiation primary response 88
NanA	neuraminidase A
NLR	Nod-like receptor
NLRP3	Nod-like receptor (NLR) family pyrin domain containing 3
NOS	nitric oxide synthase
PAF	platelet-activating factor
PAMP	pathogen-associated molecular patterns
PECAM-1	platelet endothelial cell adhesion molecule
PLA2	pneumococcal phospholipase 2
plgR	poly immunoglobin receptor
ply	pneumolysin
PM	pneumococcal meningitis
PRR	pattern recognition receptors
Psp	pneumococcal surface proteins
PspA	pneumococcal surface protein A
PVM	perivascular macrophages
RONS	reactive oxygen and nitrogen species
ROS	reactive oxygen species
TACE	tumour necrosis factor alpha converting enzyme
TGF-β	transforming growth factor-beta
TLR	Toll-like receptor
TNF	tumour necrosis factor
